# A Structural Study of *Escherichia coli* Cells Using an *In Situ* Liquid Chamber TEM Technology

**DOI:** 10.1155/2015/829302

**Published:** 2015-02-05

**Authors:** Yibing Wang, Xin Chen, Hongliang Cao, Chao Deng, Xiaodan Cao, Ping Wang

**Affiliations:** ^1^State Key Laboratory of Bioreactor Engineering, Biomedical Nanotechnology Center, East China University of Science and Technology, Shanghai 200237, China; ^2^Key Laboratory for Ultrafine Materials of Ministry of Education and Shanghai Key Laboratory of Advanced Polymeric Materials, School of Materials Science and Engineering, East China University of Science and Technology, Shanghai 200237, China; ^3^State Key Laboratory of Functional Materials for Informatics, Shanghai Institute of Microsystem and Information Technology, Chinese Academy of Sciences, 865 Changning Road, Shanghai 200050, China

## Abstract

Studying cell microstructures and their behaviors under living conditions has been a challenging subject in microbiology. In this work, *in situ* liquid chamber TEM was used to study structures of *Escherichia coli* cells in aqueous solutions at a nanometer-scale resolution. Most of the cells remained intact under electron beam irradiation, and nanoscale structures were observed during the TEM imaging. The analysis revealed structures of pili surrounding the *E. coli* cells; the movements of the pili in the liquid were also observed during the *in situ* tests. This technology also allowed the observation of features of the nucleoid in the *E. coli* cells. Overall, *in situ* TEM can be applied as a valuable tool to study real-time microscopic structures and processes in microbial cells residing in native aqueous solutions.

## 1. Introduction

High-resolution real-time observation of cells and cellular processes in their liquid environments is crucial for revealing cellular structures and functions. Electron microscopes (EMs) can easily achieve a nanometer level resolution [1]; therefore, EMs are efficient tools for observing cellular structures. However, traditional sampling procedures limit the application of EMs for real-time observation of liquid samples. Because of the high-vacuum operating conditions, extensive sample preparations (e.g., fixation, metal staining, plastic embedding and slicing [2], or freezing) are needed for biological TEM samples [3]. An in-depth understanding of cellular structures and intracellular processes requires real-time imaging of the entire biological object with high spatial resolutions in the native liquid environment. To allow real-time and fast observations of dynamic processes occurring in biological objects, the samples must be fully hydrated and nonfrozen. Environmental TEM uses a differential pump technology and therefore allows cellular studies under partially hydrated conditions [4]; however, this process is not conducted in pristine liquid environments [5, 6].

*In situ *liquid chamber TEM has been developed recently for nanostructural systems; this enables the user to image and monitor biological samples in fully hydrated environments, thus providing real-time dynamic information [7]. In this method, two ultrathin electron-transparent window chips are used to construct a liquid cell chamber; the liquid sample is sealed between these chips. The* in situ* liquid cells can be used in standard TEM instruments [8]. A variety of samples and processes of nanomaterials have been studied with this technique, including nanomaterial reactions [9] and motion [10], nanocrystal depositions [11, 12], and self-assembly of nanolipoprotein discs [13]. Biological cells have also been imaged in liquid with nanometer resolution, such as human leukocytes [14], COS7 fibroblast cells [15], yeast cells [16],* K. pneumoniae* CG43S3 cells [7], and the bacteria* Deinococcus radiodurans *(*D. radiodurans*) [17]*. E. coli* has also been investigated previously [18]. Typically, a specimen kit equipped with SiO_2_ nanomembranes is applied in TEM imaging. This kit enables the observation of living* E. coli *[7].* E. coli* has been reported to survive this TEM imaging process at 2.8 s under a 200 KeV electron beam [7]. Peckys et al. imaged* E. coli *[18] labeled with gold nanoparticles in liquid with scanning transmission electron microscopy (STEM). However, they only showed the overall morphology and viability of* E. coli* cells with limited resolution. The stability of the cells under the electron beam, the resolution, and the dynamic observation are key issues that need to be further addressed.

This study focuses on the fine structural analysis and dynamic observation of* E. coli* cells using* in situ* liquid chamber TEM. The integrity, stability, and viability of the cells under electron beams, which have not been examined in previous studies, are also examined.

## 2. Materials and Methods

*Escherichia coli *BL21(DE3) was grown in Luria-Bertani (LB) broths at 37°C for 12 h and then stored at 4°C before being used. The liquid chamber used for the* in situ* TEM was developed following a previously described procedure [9–11, 18]. In brief, silicon chips with 50 nm thick Si_3_N_4_ membranes (Ted Pella, Inc., CA, USA) were used as the electron transparent windows for the liquid chamber. The size of the windows was 0.5 × 0.5 mm. A JEOL JEM 2010 TEM (JEOL Ltd., Tokyo, Japan) was used for the* in situ* TEM and was operated under a 200 kV acceleration voltage. The beam intensity measured on the phosphorous screen is ~50 pA/cm^2^. The* E. coli* cells used for fluorescence microscopy were suspended in 0.85% NaCl after growth medium was removed. The sample was then mixed with 2x Bacterial Viability Kit stock solution with an equal volume. LIVE/DEAD BacLight Bacterial Viability Kit was from life technologies (Eugene, Oregon, USA). Briefly, a drop of 0.6 *μ*L suspension was used in liquid chamber. The liquid chamber was imaged directly by fluorescence microscopy (Olympus IX51 inverted microscope, Tokyo, Japan) with exposure time: 67.08 ms. Green fluorescence image and corresponding red fluorescence image were merged together into one picture.

## 3. Results and Discussion

**Figure 1:**
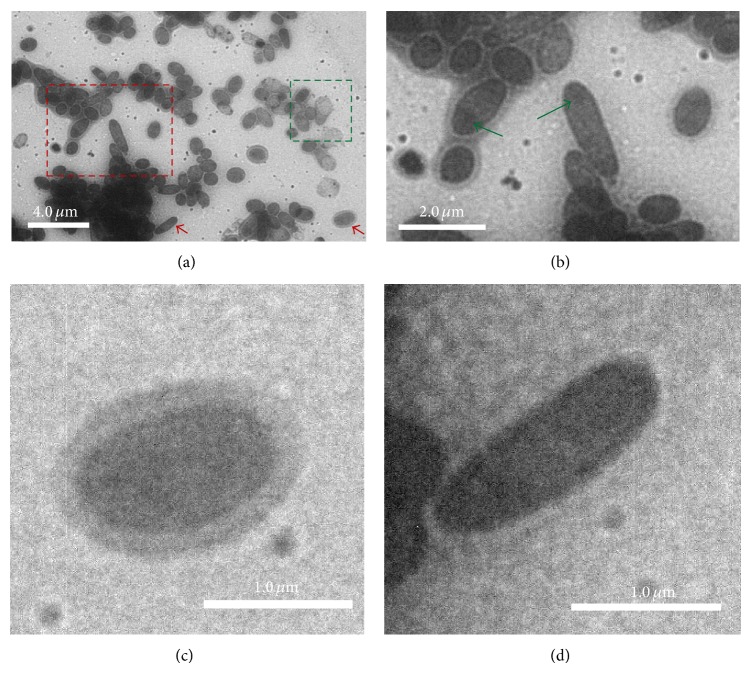
*In situ* TEM images of* E. coli* cells in the liquid chamber. (a) Larger area* in situ* TEM image. (b–d) Magnifications from (a): (b) the red rectangle labeled region in (a) in which two* E. coli* cells showed dim dark centers as highlighted by the green arrows; (c) an* E. coli *with apparent pili around the cell; (d) an* E. coli *without apparent surrounding pili. (c) and (d) displayed the cells indicated by the red arrow heads in (a).

To investigate the cellular fine structures of bacterial cells under aqueous conditions,* in situ* TEM was performed on an* E. coli* sample in the liquid chamber. Most of the cells appeared to be structurally robust against electron beam irradiation and preserved nanostructures during the TEM imaging. The* E. coli* cells in the* in situ* bright field TEM images (Figure 1(a)) were oval-rod shaped. The results are comparable to previous* in situ *liquid chamber TEM studies [7, 18]. Furthermore, as seen in Figures 1(b)–1(d), our* E. coli* images show more details than other reports in the literature, with several cellular structures detected outside of the oval-rod shaped bacterial cells and a few cells showing dim dark nucleoid centers, which are indicated by the two green arrows in Figure 1(b). The fine structures outside of the* E. coli* cells in the* in situ* TEM images may be attributable to pili, which are displayed as gray halos around some of the bacterial cells (Figure 1(c)). Almost half of the* E. coli* cells showed pili, but the other cells had no apparent pili structures (Figure 1(d)). Whether the pili were observed might be related to the biological activity and cell life cycle of the bacteria. This conforms with the property of pili. It is possible that the resolution of* in situ* TEM might not be high enough to detect additional intracellular details of the* E. coli* cells, such as a clear nucleoid. However, images have been reported with better than 1 nm and even atomic resolutions using* in situ* liquid chamber TEM, which indicates that getting enough resolution should not be an obstacle for the technology. In our* in situ* TEM images of* E. coli* cells, pili were observed clearly, which are known to have diameters less than 10 nm. Resolutions better than 10 nm have been obtained as measured from the TEM images, which is more than enough to image a clear nucleoid if it exists. Thus, the lack of details for* E. coli* is not due to the resolution limit of* in situ* TEM but may be because of the stage of the cells during the observation. The absence of low contrast in the dark centers may suggest that the nucleoids in the* E. coli* cells under the fully hydrated environment were not as condensed as those cells that were dried or processed to be used in other imaging techniques.

**Figure 2:**
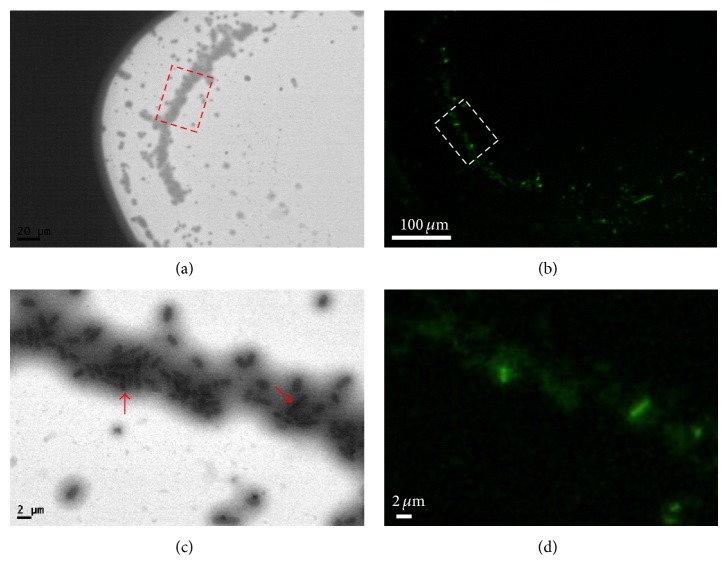
*In situ* TEM images (a, c) and corresponding fluorescence microscopy images (b, d) of* E. coli* cells in the liquid chamber after high energy beaming. The fluorescence images were obtained after TEM exposing. (c) and (d) are magnifications from squares in (a) and (b). The red arrows in (c) pointed to two cells which emitted obvious green fluorescence in (d).

With nanometer resolutions and the capability of observing cells in their fully hydrated living environments,* in situ *TEM is a more powerful tool than conventional EMs. However, the length of time that a cell can withstand the electron beam irradiation without being subjected to structural damage or cell death is also of concern. Currently, studies have only successfully irradiated cells for several seconds using* in situ* TEM, which has largely limited its applications to* in situ* monitoring of dynamic changes in the cells. In this study,* E. coli* cells demonstrated good structural stability. The cells were observed under the electron beam for extended time, and the majority of the cells did not show beam-induced structural damage. The cell viability was examined by using LIVE/DEAD BacLight Bacterial Viability Kit, and the observations are shown in Figure 2. The cells exposed under TEM high energy electron beam (Figure 2(a)) still emitted green fluorescence (which indicated alive cells) (Figure 2(b)), instead of red fluorescence (which indicated dead cells). Figures 2(c) and 2(d) show magnified versions of the images. The red arrows in Figure 2(c) pointed to two cells which emitted obvious green fluorescence as shown in Figure 2(d). The other cells also emitted green fluorescence but could not be observed clearly, possibly due to a variation of imaging depth. These results further verified the survival of the* E. coli* cells through the analysis.

**Figure 3:**
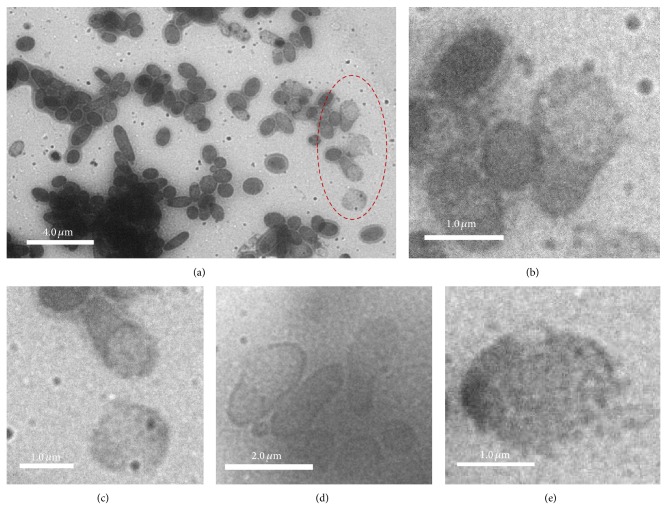
*In situ* TEM images showing the ruptured* E. coli* cells: (a) in a large area image and (b) in the magnification area from the red circular labeled region in (a) that shows a ruptured cell; (c) two ruptured cells from (a) beneath the circular region; (d) and (e) are* in situ *TEM images showing different ruptured structures.

In addition to the overall stability and integrity under the* in situ* TEM condition, several* E. coli* cells ruptured. In Figure 3(a), a few cells in the right area of the picture that are circled by red dotted lines were found to be lighter, suggesting that they were broken cells. To the right of the figure, we detected several such broken cells forming a vertical line. In the magnified image in Figure 3(b), one cell was broken from the top end. Compared to the intact cells, the ruptured cell showed a size extension at the broken site, which might partially be because of the unfolding of the cell membrane and the extruding pressure from the cytoplasm. The nonruptured end appeared to be smaller, possibly related to the loss of cytoplasm and pressure inside the cell. In Figure 3(c), in addition to the partially broken cell in the upper part, a completely broken cell is displayed in the lower part of the image. This cell showed a dim cell membrane with a larger diameter than a complete cell, and the cellular region within the membrane boundary has a lighter shade than an unbroken cell. In Figure 3(d), in addition to the cell to the right that was broken at one end, one* E. coli *cell to the left was broken from the middle, forming a pore.  Figure 3(e) shows a lytic broken cell forming membrane fragments, and as a result the outline of the oval shape of the cell was not clear. Several studies have shown that* E. coli* cells become lytic when the cell membrane is damaged [19], releasing intracellular components [20]. The phenomenon we observed could be cell lysis induced by damage to the membrane. In previous studies, pores have been suggested to form at the early stages. Additionally, the osmotic pressure across the membrane might also induce membrane rupture and cause more dramatic damage to a bacterial cell [21]. The initiation of this rupture was not clear and may not necessarily be caused by the imaging because these cells might have already broken before being examined.

**Figure 4:**
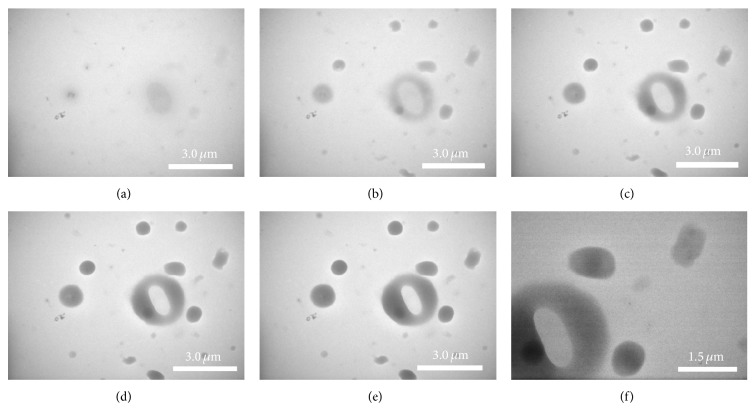
TEM images showing the process of cell damage: (a–e) show the outer ring of the oval-shaped* E. coli* cell becoming gray and surrounding gray dots appeared. (f) A magnified image taken after (e).

Although rare, a dynamic process of cell damage has also been observed using* in situ* TEM. Within a minute, an oval-shaped* E. coli* was quickly damaged. The outer ring of the oval-shaped* E. coli* cell became deeper gray, getting darker and wider with time. The image of the* E. coli* cell became brighter with time (Figures 4(a)–4(d)). In the final image (Figure 4(f)), the contour of the* E. coli* was rod-like, smaller in size, and the brightness matched the background of the picture. In Figures 4(a)–4(e), approximately eight growing dark spots were also observed. The spots might originate from the dissolution of cellular debris. One reason for such coincidental damage might be the effect of radiation from beam-sample interactions. When high energy electrons pass through an aqueous solution, the liquid system produces radicals and hydrated electrons, such as OH radicals [22, 23]. These radicals and hydrated electrons could damage the biomolecules of the cells. To analyze the effect of radicals on* E. coli* cells, 10 mg/mL glucose was added to the medium as radical scavengers. In the liquid chamber containing glucose, most of the* E. coli* cells kept their native structures without damage. This chamber showed lower red fluorescence than the liquid chamber without glucose (shown in Figure S1 in Supplementary Material available online at http://dx.doi.org/10.1155/2015/829302). This indicated less dead cells in liquid chamber with glucose.

**Figure 5:**
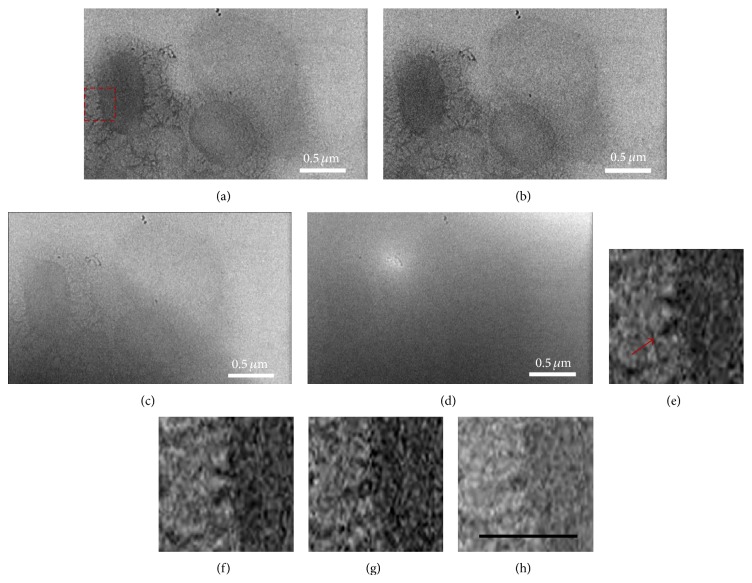
Video frames taken from the labeled region in Figure 1(a): (a) 0 s, (b) 2 s, (c) 40 s, and (d) 60 s. (e)–(h) Magnified video frames taken from the labeled region in Figure 5(a): (e) 0 s, (f) 10 s, (g) 20 s, and (h) 30 s. The arrow in (e) points to a pilus that changed shape with time. The scale bar in (h) is 0.2 *μ*m.

In the higher resolution images, pili were distinguished in the halo around the cells. Figures 5(a) and 5(b) show video frames taken from the labeled region in Figure 1(a). Partially because of the higher magnification, we obtained better images and can clearly detect the pili surrounding the cells. These pili observed in the current work resemble those previously reported using conventional imaging techniques [24, 25]. Although the samples were in an isolated chamber, the liquid flow can still occasionally be observed in the sample. The liquid flow behavior was captured in the video. In Figure 5(c) (lower right part of the image), we detected a thicker liquid layer flowing into the cell, which got darkened and blurred.  Figure 5(d) shows an image taken after the thick liquid covered the full imaging region, which produced an almost featureless picture because of the electron diffraction through the thick sample. Figures 5(e)–5(h) show magnified video frames taken from the labeled region in Figure 5(a); the arrow points to a pilus that changed shape with time. For the video of Figure 5 only, the imaging time was longer than 60 s, much longer than reported exposure time in the literature, and we did not see any structural damage to the cell. This makes a significant progress in dynamic observation of biosample using* in situ* liquid chamber TEM.

**Figure 6:**
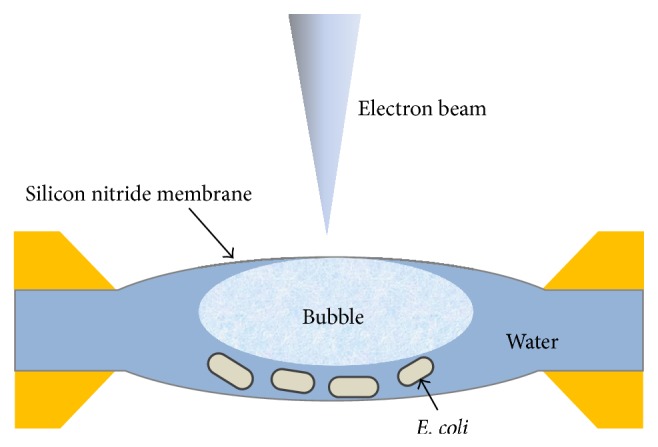
Schematic of the* in situ* liquid chamber. Liquid with* E. coli* cells was filled in chamber formed by two Si_3_N_4_ membranes. A bubble existed in the chamber, which was benefit to high-resolution images of biosamples.

Generally, people believe there are big bubbles in the* in situ* liquid chamber [26, 27], filling the majority of the space, leaving possibly only an ultrathin layer of liquid on the two windows, which was indicated in Figure 6. Thus the cells are not necessarily surrounded by liquid only but are surrounded by liquid plus vapor, which explains the good image resolution. Even when surrounded by vapor only, the surrounding vapor pressure is much larger than in an environmental TEM; thus the cells are more hydrated. Thus, it is easily understood that the liquid layer thickness can change with time due to liquid flow, resulting in the image resolution change, supporting the observation in Figures 5(a)–5(d) [18]. In addition to the motion of the pili and the liquid-cell interactions observed, TEM allows the study of various events, such as flagellar motions, nanoparticle-cell interactions, and even cell divisions. Therefore, this tool could be beneficial in numerous important studies investigating cell structures and processes.

## 4. Conclusions

In conclusion,* in situ* liquid chamber TEM was used to image* E. coli* cells in liquid environments. The* in situ* TEM results clearly displayed contours and fine cellular structures (such as pili) of the* E. coli* cells. Notably, the nucleoids in the* E. coli* cells in the fully hydrated environment appeared to be in noncondensed forms. Additionally, during our* in situ *TEM experiments, most of the* E. coli* cells were robust under the electron beam, without showing structural damage over time. The liquid chamber TEM allowed several fine structure and motion observations that were impossible with traditional TEM or SEM analyses. Liquid chamber TEM is capable of* in situ* monitoring of significant biological processes, including cell rupture and cellular structural movement. A variety of cell damage types were also observed. Notably, liquid flow behavior was observed. Additionally, the motion of the pili was recorded for the first time using the liquid chamber TEM technology. Overall, the* in situ* liquid chamber TEM is a facile and efficient tool for* E. coli* analysis. This method provides reliable studies of the cellular structures and behaviors in their native growing environments.

## Supplementary Material

The fluorescence microscopy images of *E. coli* cells in the liquid chamber with or without glucose as radical scavengers were given in Supplementary Material, in order to analyze the effect of radicals which may generate when high energy electrons pass through liquid chamber.
