# Supplementary material for: A Structural Study of Escherichia coli Cells Using an In Situ Liquid Chamber TEM Technology
Source: J Anal Methods Chem. 2015 Feb 5;2015:829302. doi: 10.1155/2015/829302 (PMC4334870; doi:10.1155/2015/829302)
Supplement: Supplementary file 1 — The fluorescence microscopy images of E. coli cells in the liquid chamber with or without glucose as radical scavengers were given in Supplementary Material, in order to analyze the effect of radicals which may generate when high energy electrons pass through liquid chamber. [file 829302.f1.pdf]

Supporting information

A structural study of *Escherichia coli* cells using an *in situ* liquid chamber TEM technology

Yibing Wang[a], Xin Chen[b,c,*], Hongliang Cao[b], Chao Deng[b], Xiaodan Cao[a], Ping Wang[a,*]

[a]*State Key Laboratory of Bioreactor Engineering, Biomedical Nanotechnology Center, East China University of Science and Technology, Shanghai 200237, P. R. China. E-mail: pwang11@ecust.edu.cn; Fax: 86-021-64250533; Tel:86-021-64250533*

[b]*Key Laboratory for Ultrafine Materials of Ministry of Education and Shanghai Key Laboratory of Advanced Polymeric Materials, School of Materials Science and Engineering, East China University of Science and Technology, Shanghai 200237, P. R. China. E-mail: xinchen73@ecust.edu.cn; Fax:86-021-64253582; Tel:86-021-64253582*

[c]*State Key Laboratory of Functional Materials for Informatics, Shanghai Institute of Microsystem and Information Technology, Chinese Academy of Sciences, 865 Changning Road, Shanghai 200050, P. R. China.*

E-mail: xinchen73@ecust.edu.cn; Fax:86-021-64253582; Tel:86-021-64253582

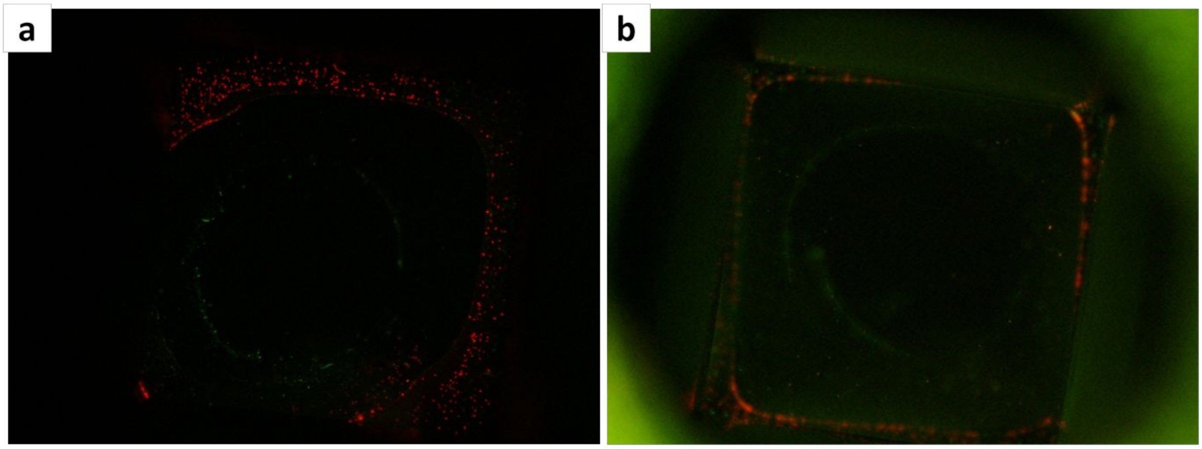

Fig. S1 The fluorescence microscopy images of *E. coli* cells in the liquid chamber without (a) or with (b) glucose. The images were got after TEM exposing and at the same exposure time.
